# Hb Knossos (HBB: c.82G > T), β-globin CD 5 (−CT) (HBB: c.17_18delCT) and δ-globin CD 59 (−a) (HBD: c.179delA) mutations in a Syrian patient with β-thalassemia intermedia

**DOI:** 10.1186/s12887-019-1435-5

**Published:** 2019-02-18

**Authors:** Faten Moassas, Mohamad Sayah Nweder, Hossam Murad

**Affiliations:** Molecular Biology and Biotechnology Department, Human Genetics Division, Atomic Energy Commission of Syria, P.O. Box 6091, Damascus, Syria

**Keywords:** Hb Knossos, δ-Thalassemia (δ-thal), β-Thalassemia (β-thal), Mutations, Syria

## Abstract

**Background:**

Beta thalassemia (β-thal) is an inherited hemoglobin disorder characterized by reduced synthesis of the hemoglobin that results in microcytic hypochromic anemia. β-Thalassemia intermedia (TI) is a clinical term of intermediate gravity between the carrier state and β-thalassemia major (β -TM).

**Case presentation:**

We describe a 12-year-old male proband originating from Al-Quneitra province - southwest Syria. Hematological investigations revealed, pallor and anemia (Hb 9 g/dl). The mean cell volume (MCV) 64 fL; mean cell hemoglobin (MCH) 21.8 pg. Capillary electrophoresis (CE) electropherogram revealed low level of Hb A1 (36.2%), high level of Hb F (62.2%) and low level of Hb A2 (1.6%). The proband requires blood transfusion occasionally. Direct DNA sequencing and Polymerase chain reaction-restriction fragment length polymorphism (PCR/RFLP) for mutations detection were used. The molecular analysis revealed the presence of rare β^+^ Hb Knossos codon 27 (G > T) (HBB: c.82G > T) variant associated with β^0^ codon 5 [−CT] (HBB: c.17_18delCT) mutation in beta-globin (β-globin) gene and δ^0^ codon 59 [−A] (HBD: c.179delA) mutation in delta-globin (δ-globin) gene. The proband tested negative for the common deletional forms of alpha thalassemia (α-thal). Polymorphism of the Xmn-I locus (HBG2: c.-211C > T) revealed that the proband had a homozygous [TT] for Xmn-1 locus.

**Conclusions:**

To our knowledge, this is the first report of beta thalassemia intermedia due to combination of Hb Knossos /codon 5 [−CT] associated with δ^0^ codon 59 [−A] in Syrian patient. On the other hand, in Syria, β-thal carriers who have low level of Hb A2 due to decreased δ-chain production, different δ-thal gene mutations must be screened to avoid the failure diagnosis of β-thal disease.

## Background

Thalassemia (thal) is one of the most common inherited blood disorder in the world. This disease caused by reduced or/and absent synthesis of the globin chains of hemoglobin (Hb), which leading to imbalance of the globin chains [1, 2]. Beta-thalassemia (β-thal) is one of the major types of thalassemia and it results from decrease in lack of beta-globin (β-globin) chain production [3]. Thalassemia intermedia (TI) or non-transfusion-dependent thalassemia (NTDT) is a moderate clinical form of the β-thal disease.

It has a broad clinical spectrum, spanning in severity from asymptomatic thalassemia minor to transfusion-dependent thalassemia major (TM) phenotype [4]. Thalassemia intermedia can result from the inheritance of one or two β-thal alleles [5–8]. On the other hand, no clinical significance have been observed for delta-globin (δ-globin) gene mutations, but this gene has important relevance for the screening of β-thal carriers [9]. Hemoglobin A2 (Hb A2), is a minor adult hemoglobin, its levels ranged between (2–3.2%) of the total circulating haemoglobin in healthy adults [10]. The increase in Hb A2 level more than borderline levels is the most important parameter for the identification of thalassemia carriers [2]. The presence of δ-thal mutation, however, interferes with this typical β-thal phenotype, affecting population screening programs for β-thal carriers. Delta/beta-thalassemia (δβ-thal) is as a consequences of a deletion in both the delta and beta genes on chromosome 11. This deletion leads to increase of production of gamma globin (*y*-globin) gene, which increases the amount of fetal hemoglobin (Hb F). The δβ-thal heterozygotes clinically display characteristics of thalassemia minor. However, homozygous δβ-thal state could appear a clinical description of thalassemia intermedia (TI) with a mild anemia [11]. Some genome-wide association studies have reported that there are at least three major loci that play a major role in increasing Hb F levels [12]. One of them, is the − 158 C > T (HBG2: c.-211C > T) in the promoter gene Gamma 2 (locus XmnI). This locus simultaneously has an influence on up to 20–50% of Hb F variation in patients with β-thal or in healthy adults [13].

Here, we report a β-thal affected proband with low level of Hb A2 who had point mutation in codon 5 [−CT] combined with Hb Knossos codon 27 (G > T) on the β-globin gene associated with codon 59 [−A] mutation on the δ-globin gene. To the best of our knowledge, this is the first report which described the Hb Knossos /codon 5 [−CT] genotype associated with δ-thal mutations in Syrian patient.

### Case presentation

12-year-old male proband, was referred to our center AECS- in Damascus for confirmation of his affected status for β-thalassemia. The parents were non-consanguineous. His history revealed, pallor and anemia. The electropherogram revealed low level of (Hb A1) 36.2%, high level of (Hb F) 62.2% and low level of (Hb A2) 1.6%, for that, δ-globin gene variant was suspected. His father had a classical clinical picture of β-thalassemia trait. His mother had normal indices but with reduced (Hb A2 levels) 1.9%, all hematological data were summarized in Table 1. The parents had never been transfused, while the proband requires blood transfusion occasionally.

**Table 1:** The hematological and molecular data of the family

Parameters	Proband’s Father	Proband’s Mother	Proband
Sex-age (years)	M-40	F-38	M-12
Hb (g/dL)	11.2	14	9
RBC (10^12^/L)	5.4	5.5	4.2
MCV (fL)	60.42	83	64
MCHC (g/dl)	31.5	32.2	33.9
MCH (pg)	19.35	27.3	21.8
RDW-CV (%)	14.9	15.6	18
Hb A1 (%)	93.2	98.1	36.2
Hb A2 (%)	3.8	1.9	1.6
Hb F (%)	3	0	62.2
α Genotype	αα/αα	αα/αα	αα/αα
β Genotype	β ^A^/β ^codon 5[−CT]^	β ^A^/β ^Hb Knossos^	β ^codon 5[−CT]^ /β ^Hb Knossos^
δ Genotype	δ ^A^/δ ^A^	δ ^A^/δ ^Codon 59 (−A)^	δ ^A / Codon 59 (−A)^

*RBC* red blood cell count, *Hb* hemoglobin, *MCV* mean corpuscular volume, *MCH* mean corpuscular *Hb*, *RDW-CV* RBC distribution width- coefficient of variation.

To investigate the high level of Hb F in the proband, the XmnI restriction site at − 158 position of the ^G^γ-gene was done. Hematological parameters of the parents and proband were obtained with an automated differential cell counter (ABX Micros ES60; HORIBA ABX SAS, Montpellier, France). Capillary Hemoglobin electrophoresis (Hb) analysis were measured using Capillarys 2 system (Sebia, Lisses, France) system.

After obtaining informed consent, genomic DNA was isolated from peripheral blood from the parents and proband using the QIAamp DNA Blood Mini kit (Qiagen, Germany) according to the manufacturer’s instructions. Purified gDNA was run on a 0.8% agarose gel. The quality and quantity of the DNA was determined spectrophotometrically (NanoVue™; GE HealthCare, Freiburg, Germany).

Direct DNA sequencing of the entire human HBB and HBD genes was done on an ABI PRISM 310-DNA Analyzer (Applied Biosystem, Foster City, CA, USA) as previously reported [14, 15]. The genotyping of HBB gene was determined by polymerase chain reaction (PCR). The suitable primers were used for three exons of β-globin gene including the promoter, first intron, 5’ and 3’ untranslated region (UTR) sequences as previously reported [16]. For HBD gene, two specific primer sets were designed for Ex 1& 2 and Ex 3 including their flanking regions on the δ-globin gene as previously reported [17]. Reverse hybridization assay (α-Globin StripAssay® 4–160; ViennaLab Diagnostics Gmb Vienna, Austria) which covers 21 of α-thal mutations was used according to the manufacturer’s instructions. Detection of Xmn-I locus was performed with RFLP-PCR technique with specific primers and restriction enzyme Xmn-I [13].

**Fig. 1:**
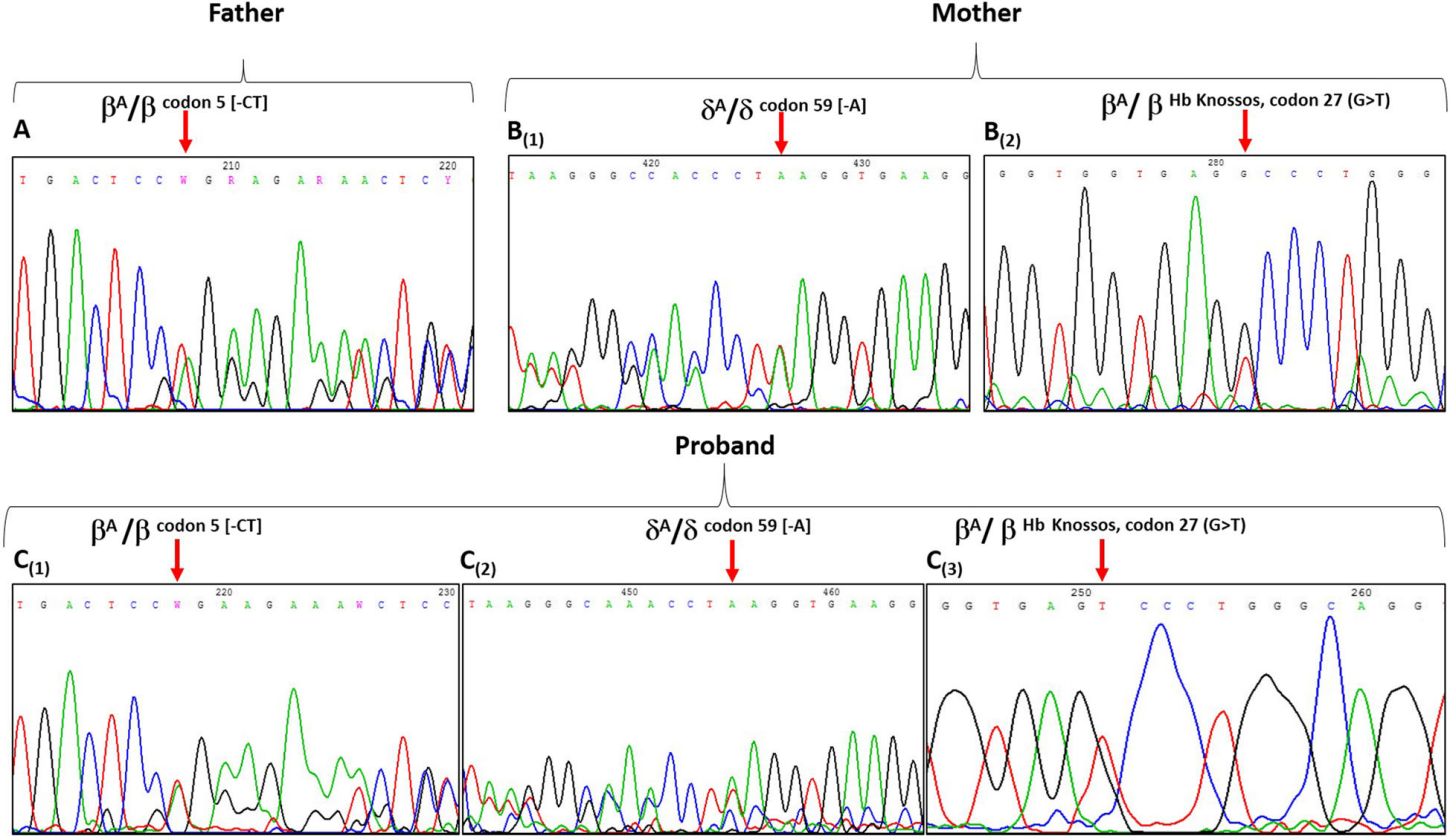
Direct sequencing analysis revealed the PCR fragment on the δ-globin and β-globin genes. (A), (C_1_) the arrows indicates the [−CT] deletion at codon 5 in the β-globin gene for the father and the proband respectively; (B_1_), (C_2_) the arrows indicates the [−A] deletion at the codon 59 in the δ-globin gene for the mother and the proband respectively; (B_2_), (C_3_) the arrows indicates the Hb Knossos substitution at the codon 27 in the β-globin gene for the mother and the proband respectively

In this case, the blood and physical examination of the proband showed that, and he had anemia and pallor, and he was affected by delta and beta thalassemia. Hematological and molecular data for the family were described in Table 1. Direct DNA sequencing for β-globin and δ-globin genes shown in Fig. 1. The father had the β^0^ Codon 5 [−CT] mutation in heterozygous state, whereas, the mother presented the β^+^ Hb Knossos codon 27 (G > T) mutation with δ^0^ codon 59 [−A] mutation both in heterozygous state, thus resulting in a low level of Hb A2 (1.9%) (Fig. 1).

The Molecular analysis of the proband showed that, he had inherited the β^0^ codon 5 [−CT] mutation from his father, and had inherited the β^+^ Hb Knossos codon 27 (G > T) mutation and the δ^0^ codon 59 [−A] mutation from his mother, so, a low level of Hb A2 (1.6%) was also observed (Fig. 1). On the other hand, the results of the α-thal test in the proband revealed that none of the common deletional forms were present.

The result of PCR/RFLP of the Xmn-I locus at − 158 to the ^G^훾-globin gene indicated that, the homozygosity [TT] genotype in the proband was observed.

## Discussion and conclusions

Hb Knossos is a rare Hb variant in the world, it was first described in a Greek family [18]. The β^+^ Hb Knossos, codon 27 (G > T) (HBB: c.82G > T, p.Ala28Ser) activates a cryptic splice site in the β-globin gene which competes with the normal splice site thereby resulting in reduced production of Hb Knossos mRNA [19]. It is described to produce the classical phenotype of β-thal intermedia in association with β^0^-thal trait. Also, it has been reported in combination with different β-thal mutations like IVS-I-1 (G > A) [20] and IVS-I-110 (G > A) [21] causing a moderate phenotype, whereas its association with the IVS-II-745 (C > G) resulted in a major phenotype [22]. The β^0^ Codon 5 [−CT] mutation was well-known to complete inhibition of β-chain synthesis through the formation of a premature termination signal at codon 21. In our case, the combination of β^+^ Hb Knossos with β^0^ codon 5 [−CT] mutation is reported for the first time, and it leads to β-IT phenotype.

The δ-globin gene mutations have no clinical implication. However, the co-inheritance of δ-globin gene variant with β-thal may camouflage the β-thal carrier status by decreasing the Hb A2 levels [11, 23, 24]. The codon 59 [−A] mutation is one of the rare δ^0^-globin gene mutation, it deletes a single A in codon 59 leading to premature termination in codon 60. Hb Knossos mutation was reported to be linked to δ^0^-globin gene codon 59 [−A] mutation in the majority of North African and Mediterranean countries [25–27]. This combination correlated to normal or borderline red blood cell indices and also with low Hb A2 levels [25, 28]. In this study, the δ^0^ codon 59 [−A] mutation was observed for the first time in a Syrian family, and it was associated with the β^+^ Hb Knossos mutation for the proband and mother. In addition, a coinheritance of β^0^ codon 5 [−CT] mutation with δ^0^ codon 59 [−A] mutation was observed for the proband. Two mutations shown to be inherited *in trans*, the proband inherited the β^0^ codon 5 [−CT] mutation from his father and the δ^0^ codon 59 [−A] mutation from his mother. On the other hand, the level of Hb F was (62.2%) for the proband, and the genotype of Xmn-I polymorphism was homozygous [TT]. This factor may be contributed to produce a high level of Hb F as previously reported [29, 30].

In conclusion, we present here a case of rare β^+^ Hb Knossos codon 27 (G > T) variant associated with β^0^ codon 5 [−CT] mutation in β-globin gene and δ^0^ codon 59 [−A] mutation in δ-globin gene which were found in Syrian male proband for the first time in a Syrian family. However, As Syria is one of the countries where β-thal is prevalent, δ-thal mutations should be investigated at the β-thal carriers when we have low level of Hb A2, due to interactions between these haemoglobinopathies which can failing to diagnose the β-thalassemia carriers.

## Abbreviations

Hb F: Hemoglobin F
Hb: Hemoglobin
MCH: Mean cell hemoglobin
MCHC: Mean cell hemoglobin concentration
MCV: Mean cell volume
PCR: Polymerase chain reaction
RBC: Red blood cell
RDW: Red cell distribution width

## References

[1] Weatherall DJ, Clegg JB (1996). Thalassemia--a global public health problem. Nat Med.

[2] Galanello R, Origa R (2010). Beta-thalassemia. Orphanet J Rare Dis.

[3] Zhang J, Yan J, Zeng F (2018). Recent Progress on genetic diagnosis and therapy for β-thalassemia in China and around the world. Hum Gene Ther.

[4] Asadov C, Alimirzoeva Z, Mammadova T, Aliyeva G, Gafarova S, Mammadov J (2018). β-Thalassemia intermedia: a comprehensive overview and novel approaches. Int J Hematol.

[5] Vinciguerra M, Passarello C, Cassara F, Leto F, Cannata M, Calvaruso G, Renda D, Maggio A, Giambona A (2018). Coheredity of a new silent mutation: c.-29G>T, with a severe beta-thal mutation in a patient with beta-thalassemia intermediate. Int J Lab Hematol.

[6] Aliyeva G, Abdulalimov E, Asadov C, Mammadova T, Gafarova S, Guliyeva Y (2018). First report of beta-thalassemia intermedia in a patient compound heterozygous for −92 (C>T) and codons 36/37 (−T) mutations. Hemoglobin.

[7] Agapidou A, King P, Ng C, Tsitsikas DA (2018). Double heterozygocity for hemoglobin C and beta thalassemia dominant: a rare case of thalassemia intermedia. Hematol Rep.

[8] Kelkar AJ, Moses A (2017). Thalassemia intermedia phenotype resulting from rare combination of c.46delT [Codon15 (−T)] mutation of beta globin gene and HPFH3. Clin Case Rep.

[9] Pavlou E, Phylactides M, Kyrri A, Kalogerou E, Makariou C, Georgiou I, Delta-thalassemia in Cyprus KM (2006). Hemoglobin.

[10] Goonasekera HW, Paththinige CS, Dissanayake VHW (2018). Population screening for Hemoglobinopathies. Annu Rev Genomics Hum Genet.

[11] Mansoori H, Asad S, Rashid A, Karim F (2016). Delta beta thalassemia: a rare hemoglobin variant. Blood Res.

[12] Lettre G (2012). The search for genetic modifiers of disease severity in the beta-hemoglobinopathies. Cold Spring Harb Perspect Med.

[13] Rujito L, Basalamah M, Siswandari W, Setyono J, Wulandari G, Mulatsih S, Sofro AS, Sadewa AH, Sutaryo S (2016). Modifying effect of XmnI, BCL11A, and HBS1L-MYB on clinical appearances: a study on beta-thalassemia and hemoglobin E/beta-thalassemia patients in Indonesia. Hematol Oncol Stem Cell Ther.

[14] Murad H, Moassas F, Jarjour R, Mukhalalaty Y, Al-Achkar W (2014). Prenatal molecular diagnosis of beta-thalassemia and sickle cell anemia in the Syrian population. Hemoglobin..

[15] Murad H, Moasses F, Dabboul A, Mukhalalaty Y, Bakoor AO, Al-Achkar W, Jarjour RA (2018). Geographical distribution of beta-globin gene mutations in Syria. Hematology.

[16] Yassin MM, Sirdah MM, Al Haddad RM, Lubbad AH, Al-Yazji MS (2013). Genotype-phenotype characteristics of β thalassemia children in the Gaza strip, Palestine. J Genet Disor Genet Rep.

[17] Liu N, Xie XM, Zhou JY, Li R, Liao C, Li DZ (2013). Analysis of delta-globin gene mutations in the Chinese population. Hemoglobin.

[18] Fessas P, Loukopoulos D, Loutradi-Anagnostou A, Komis G (1982). 'Silent' beta-thalassaemia caused by a 'silent' beta-chain mutant: the pathogenesis of a syndrome of thalassaemia intermedia. Br J Haematol.

[19] Orkin SH, Antonarakis SE, Loukopoulos D (1984). Abnormal processing of beta Knossos RNA. Blood.

[20] Gurgey A, Balkan H, Irken G, Gumruk F, Altay S, Kalaycioglu A, Oner C, Oner R (1997). Compound heterozygosity for hemoglobin Knossos [alpha 2 beta 2 27 (B9) ala-Ser] and IVS I-1 mutation. Turk J Pediatr.

[21] Altay C, Gurgey A (1990). Beta-thalassemia intermedia in Turkey. Ann N Y Acad Sci.

[22] Keser I, Manguoglu E, Kayisli O, Yesilipek A, Luleci G (2007). Combination of Hb Knossos [cod 27 (G-T)] and IVSII-745 (C-G) in a Turkish patient with beta-thalassemia major. Genet Test.

[23] Sun M, Lou J, Zhao Y, Liu Y. Molecular and Hematological Characterization of Two Novel delta-Globin Gene Mutations Found in Chinese Individuals. Hemoglobin. 2018;42:132-134. 10.1080/03630269.2018.1458628 29722583

[24] Velasco-Rodriguez D, Alonso-Dominguez JM, Gonzalez-Fernandez FA, Villarrubia J, Ropero P, Martinez-Nieto J, de la Fuente F, Guillen R, Acedo N, Seri C (2014). Cava F. Deltabeta-thalassemia trait: how can we discriminate it from beta-thalassemia trait and iron deficiency anemia?. Am J Clin Pathol.

[25] Sahli CA, Bibi A, Ouali F, Siala H, Fredj SH, Othmani R, Ouenniche F, Cheour M, Fitouri Z, Becher SB, Messaoud T (2012). δ0-thalassemia in cis of β Knossos globin gene: first homozygous description in thalassemia intermedia Libyans and first combination with codon 39 (C → T) in thalassemia intermedia Tunisian patients. Clin Chem Lab Med.

[26] Olds RJ, Sura T, Jackson B, Wonke B, Hoffbrand AV, Thein SL (1991). A novel delta 0 mutation in cis with Hb Knossos: a study of different genetic interactions in three Egyptian families. Br J Haematol.

[27] Oner R, Birben E, Acar C, Oner C, Kara A, Gumruk F, Gurgey A, Altay C (2000). Molecular analysis of turkish beta-thalassemia heterozygotes with normal Hb A2 levels. Hemoglobin.

[28] Nasouhipur H, Banihashemi A, Youssefi Kamangar R, Akhavan-Niaki H (2014). Hb Knossos: HBB:c.82G>T associated with HBB:c.315+1G>a Beta zero mutation causes thalassemia intermedia. Indian J Hematol Blood Transfus.

[29] Aditya R, Verma IC, Saxena R, Kaul D, Khanna VK (2006). Relation of Xmn-1 Polymorphism & Five Common Indian Mutations of Thalassaemia with phenotypic presentation in b-Thalassaemia. JK Sci.

[30] Pereira C, Relvas L, Bento C, Abade A, Ribeiro ML, Manco L (2015). Polymorphic variations influencing fetal hemoglobin levels: association study in beta-thalassemia carriers and in normal individuals of Portuguese origin. Blood Cells Mol Dis.

